# Copper interferes with selenoprotein synthesis and activity

**DOI:** 10.1016/j.redox.2020.101746

**Published:** 2020-10-07

**Authors:** Maria Schwarz, Kristina Lossow, Katja Schirl, Julian Hackler, Kostja Renko, Johannes Florian Kopp, Tanja Schwerdtle, Lutz Schomburg, Anna Patricia Kipp

**Affiliations:** a Department of Molecular Nutritional Physiology, Institute of Nutritional Sciences, Friedrich Schiller University Jena, Jena, 07743, Germany; b TraceAge-DFG Research Unit on Interactions of Essential Trace Elements in Healthy and Diseased Elderly, Potsdam-Berlin-Jena, Germany; c German Institute of Human Nutrition, Nuthetal, 14558, Germany; d Institute for Experimental Endocrinology, Charité - University Medical School Berlin, Berlin, 13353, Germany; e German Federal Institute for Risk Assessment (BfR), Berlin, Germany; f Department of Food Chemistry, Institute of Nutritional Science, University of Potsdam, Nuthetal, 14558, Germany

**Keywords:** Selenium, Copper, Selenoprotein synthesis, Glutathione peroxidase, Thioredoxin reductase

## Abstract

Selenium and copper are essential trace elements for humans, needed for the biosynthesis of enzymes contributing to redox homeostasis and redox-dependent signaling pathways. Selenium is incorporated as selenocysteine into the active site of redox-relevant selenoproteins including glutathione peroxidases (GPX) and thioredoxin reductases (TXNRD). Copper-dependent enzymes mediate electron transfer and other redox reactions. As selenoprotein expression can be modulated e.g. by H_2_O_2_, we tested the hypothesis that copper status affects selenoprotein expression. To this end, hepatocarcinoma HepG2 cells and mice were exposed to a variable copper and selenium supply in a physiologically relevant concentration range, and transcript and protein expression as well as GPX and TXNRD activities were compared. Copper suppressed selenoprotein mRNA levels of GPX1 and SELENOW, downregulated GPX and TXNRD activities and decreased UGA recoding efficiency in reporter cells. The interfering effects were successfully suppressed by applying the copper chelators bathocuproinedisulfonic acid or tetrathiomolybdate. In mice, a decreased copper supply moderately decreased the copper status and negatively affected hepatic TXNRD activity. We conclude that there is a hitherto unknown interrelationship between copper and selenium status, and that copper negatively affects selenoprotein expression and activity most probably via limiting UGA recoding. This interference may be of physiological relevance during aging, where a particular shift in the selenium to copper ratio has been reported. An increased concentration of copper in face of a downregulated selenoprotein expression may synergize and negatively affect the cellular redox homeostasis contributing to disease processes.

## Highlights

• Copper suppresses selenoprotein mRNA levels of GPX1 and selenoprotein W.
• Copper inhibits GPX and TXNRD activities.
• Copper decreases UGA recoding efficiency.
• Copper effects on TXNRD activity also take place in the liver of mice.

## Graphical abstract

**Figure:**
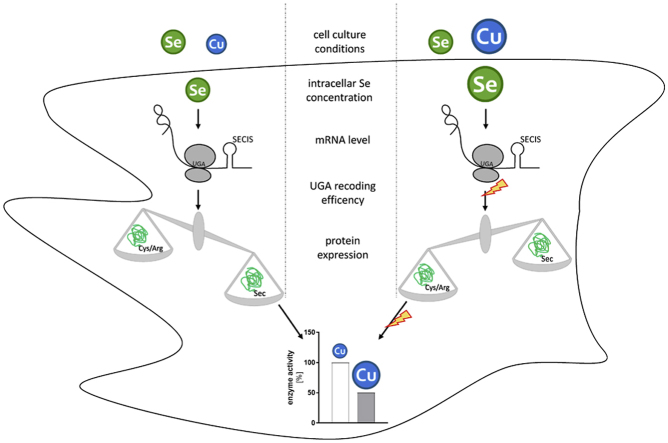

## Abbreviations

Atox1: antioxidant protein 1
BCS: bathocuproinedisulfonic acid
BSO: buthionine-sulfoximine
CCS: Cu chaperone for superoxide dismutase 1
Ctr1: Cu transporter 1
Cu: copper
DIO: deiodinase
DMT1: divalent metal-ion transporter 1
DTNB: 5,5′-dithio-bis-(2-nitrobenzoic acid)
eEFSec: Sec-specific translation elongation factor
GCL: glutamate-cysteine ligase
GSH: glutathione
GR: glutathione reductase
MT: metallothionein
MTF-1: metal regulatory transcription factor 1
MTT: thiazolyl blue tetrazolium bromide
NQO1: NAD(P)H quinone dehydrogenase 1
Nrf2: nuclear factor erythroid 2 p45-related factor 2
PSTK: O-phosphoseryl-tRNA kinase
RT: room temperature
Se: selenium
Sec: selenocysteine
SECIS: Sec insertion sequence
SeMet: selenomethionine
Sephys2: selenophosphate synthetase 2
SepSecs: Sep (O-phosphoserine) tRNA:Sec tRNA synthase
SerS: seryl-tRNA synthetase
SOD1: superoxide dismutase 1
TE: trace element
TNB: 2-nitro-5-thiobenzoic acid
TTM: tetrathiomolybdate
TXRF: total reflection X-ray fluorescence

## 1 Introduction

The biological functions of the essential trace element (TE) selenium (Se) have been attributed primarily to selenoproteins. In the human genome, 25 genes encode for selenoproteins [1]. The best characterized selenoprotein families are the glutathione peroxidases (GPXs), the thioredoxin reductases (TXNRDs), and the deiodinases (DIOs). GPXs and TXNRDs are important modulators of the cellular redox homeostasis by either catalyzing the glutathione (GSH)-dependent reduction of hydroperoxides or NADPH-dependent reduction of thioredoxins and several further substrates, respectively. Selenoprotein P (SelenoP) comprises almost 50% of total plasma Se and transports Se from liver to peripheral tissues [2]. Besides these, the function of several further selenoproteins are still not entirely understood, however, almost all of them appear to be involved in maintaining the cellular redox homeostasis. This holds especially true for selenoproteins such as selenoprotein H (SelenoH) and SelenoW which contain selenocysteine (Sec) as part of a CXXU motif, indicating that they are putative oxidoreductases. In addition, SelenoW has been shown to act in an antioxidant manner after its glutathionylation [3]. During selenoprotein synthesis, Se is cotranslationally incorporated as Sec which is encoded by the base triplet UGA. The specific Sec tRNA^[Ser]Sec^ becomes first aminoacylated with serine which is phosphorylated, accordingly. Both steps are catalyzed by seryl-tRNA synthetase (SERS) and O-phosphoseryl-tRNA kinase (PSTK), respectively. The selenophosphate synthetase 2 (SEPHS2), which also belongs to the group of selenoproteins generates monoselenophosphate, which is then used by Sep (O-phosphoserine) tRNA:Sec tRNA synthase (SEPSECS) to form selenocysteyl-tRNA^[Ser]Sec^. To initiate Sec incorporation rather than termination of protein synthesis, selenoprotein mRNAs contain a special Sec insertion sequence (SECIS) element in their 3’ untranslated region. For efficient translation of UGA to Sec, additional factors such as the Sec-specific translation elongation factor (EEFSEC) are needed (overview in Ref. [2]).

The selenoprotein synthesis can be modulated at different levels. The best characterized principle is based on the efficiency of Sec incorporation affecting selenoprotein synthesis mainly at the translational level. In case of Se deficiency, expression levels of favored selenoproteins, namely housekeeping selenoproteins such as TXNRD1, TXNRD2, and GPX4 are maintained while expression levels of so-called stress-responsive selenoproteins e.g. GPX1, SELENOH, and SELENOW are rapidly decreased. This is also called hierarchy of selenoproteins [2]. In addition, drugs such as the aminoglycoside geneticin (G418) can induce misinterpretation of the UGA codon, primarily under Se deficiency, leading to increased rates of dysfunctional variants of selenoproteins [[4], [5], [6]]. Besides the translational regulation, selenoprotein expression can be additionally modulated at the transcriptional level. A prominent example relates to the activation of nuclear factor erythroid 2 p45-related factor 2 (Nrf2) by sulforaphane, positively affecting GPX2 and TXNRD1 expression [7,8]. Besides sulforaphane, e.g. hydroperoxides contribute to selenoprotein expression and modulate read-through efficiency [9]. These examples indicate that redox-responsive transcription factors and the cellular redox homeostasis synergistically affect selenoprotein expression at different molecular levels.

Besides factors that directly impact the cellular redox homeostasis, other mechanisms may contribute in a more indirect manner. This includes the essential TE copper (Cu), which is a cofactor of antioxidant enzymes such as superoxide dismutase 1 (SOD1) [10], but at higher concentrations could also contribute as free ion to the generation of reactive oxygen species [11]. Furthermore, Cu is able to oxidize free thiol groups and to modulate the cellular redox homeostasis [12]. Thus, Cu metabolism and flux have to be strictly controlled for which a multitude of mechanisms exist. Cu is mainly taken up via the high-affinity Cu transporter 1 (Ctr1) [13], and to a lesser extent by Ctr2 [14]. The relevance of the divalent metal-ion transporter 1 (DMT1) for Cu transport is controversially discussed [15,16]. Intracellular Cu is bound to chaperones, which transport Cu to the target proteins [17]. One of these is the Cu chaperone for superoxide dismutase 1 (CCS) [18], which is upregulated when Cu levels are low [19]. Antioxidant protein 1 (Atox1) transfers Cu to ATP7A and ATP7B, essential for Cu export [17]. Further molecules that bind intracellular Cu and thus avoid free ions and/or discharge excessive Cu, are GSH and metallothioneins (MTs) [[20], [21], [22]]. In rodents, Cu deficiency led to a decreased activity of GPXs [[23], [24], [25]]. In addition, Cu is able to reverse selenite-induced cytotoxicity in chicks [26] and in HT29 cells [27]. Based on these studies it is tempting to speculate that Cu interferes with the Se homeostasis. Here we address whether low, adequate or supplemented concentrations of Se and Cu modulate their metabolism *in vitro* and *in vivo*. To this end, we analyzed TE concentrations, gene and protein expression of Se- and Cu-dependent enzymes, and enzyme activities of the selenoproteins GPX and TXNRD in relation to changes in Cu status.

## 2 Material and methods

### 2.1 Mouse experiment

Male C57BL/6Jrj mice were housed in polycarbonate cages on a 12:12 h light:dark schedule with constant room temperature (RT, 22 °C) and humidity (55%). After weaning, at the age of 3 weeks, mice received a torula yeast-based Se-deficient diet (modified C1045, Altromin, Lage, Germany) additionally low in Cu and Mn as well as Na (Table 1). For all animals, the deionized drinking water was enriched with Mn and Na, subsequently resulting in 100 ppm and 500 ppm, respectively. Cu and Se were either supplied at suboptimal (no fortification) or adequate (fortification of drinking water, finally 6 ppm and 0.15 ppm, respectively) concentrations. The supply with TEs was weekly adapted to group-specific water and food consumption of the animals to reach the final TE concentration of interest. For supplementation, CuSO_4_ (Sigma-Aldrich/Merck, Darmstadt, Germany), MnCl_2_ (Sigma-Aldrich/Merck), NaCl (Sigma-Aldrich/Merck), and Na_2_SeO_3_ (Thermo Fisher Scientific, Waltham, USA) were employed. The intervention lasted for eight weeks, in which food and water were offered *ad libitum*. Finally, mice were anesthetized with isoflurane (Isothesia, Henry Schein, Hamburg, Germany) and blood was collected by cardiac puncture. Serum was obtained after full coagulation at RT and centrifugation for 10 min (3000×*g*, 4 °C). Organs were surgically dissected and immediately frozen. All animal procedures were approved and conducted following national guidelines of the Ministry of Environment, Health and Consumer Protection of the federal state of Brandenburg, Germany (permission number 2347-44-2017) and institutional guidelines of the German Institute of Human Nutrition Potsdam-Rehbruecke.

**Table 1:** Nutrient requirement of mice [28] and TE content of the diet and drinking water [ppm].

TE	Requirement	Diet	Fortification of drinking water	Final TE supply			
				-Se/-Cu	-Se/+Cu	+Se/-Cu	+Se/+Cu
Cu	6.00	1.60	4.40	1.60	6.00	1.60	6.00
Mn	10.0	8.84	91.2	100	100	100	100
Se	0.15	0.02	0.13	0.02	0.02	0.15	0.15
Na	500	194	306	500	500	500	500

### 2.2 Cell culture

The human hepatocellular carcinoma cell line HepG2 (ACC 180 German Collection of Microorganisms and Cell Cultures (DSMZ)) and the human colorectal adenocarcinoma cell line HT-29 (ACC 299 DSMZ) were cultured in Roswell Park Memorial Institute 1640 media (RPMI; ThermoFisher Scientific) supplemented with 10% (v/v) fetal calf serum (FCS, Sigma-Aldrich/Merck), 1% (v/v) penicillin-streptomycin (P/S; ThermoFisher Scientific), and 1% (v/v) GlutaMAX™ (ThermoFisher Scientific) under standard culture conditions (37 °C, 5% CO_2_). Se and Cu are exclusively supplied by the FCS to culture media, resulting in low basal concentrations of 5 nM and 200 nM, respectively. Unless otherwise specified, cells were incubated with 50 nM sodium selenite (99%, Honeywell FlukaTM, Fisher Scientific) or 200 nM selenomethionine (SeMet; Sigma-Aldrich/Merck) and increasing concentrations (25, 50, and 100 μM) of CuSO_4_ (Sigma-Aldrich/Merck) from the time point of seeding to harvesting 72 h later. When treated with chelators, 400 μM bathocuproinedisulfonic acid (BCS, Sigma-Aldrich) or 75 μM tetrathiomolybdate (TTM, Sigma-Aldrich) were added to the culture medium 24 h before harvesting the cells. For the wash-out experiment, Cu-loaded cells (72 h of incubation) were either left without Cu (-Cu), received Cu super-depletion (-Cu, +BCS), or further Cu treatment (+Cu) for up to 120 h. Cell pellets were frozen in liquid nitrogen and stored at -80 °C until further procedure.

### 2.3 Cell viability assay

For the MTT assay, cells were seeded in 96-well plates. After 72 h of incubation with Se and Cu, 20 μl of 5 mg/ml thiazolyl blue tetrazolium bromide (MTT; Sigma-Aldrich) was added to the media. After 3 h, media were discarded followed by a 10 min shaking step with 5% (v/v) formic acid (Carl Roth, Karlsruhe, Germany) in 100% isopropanol (Carl Roth) to dissolve the obtained formazan crystals. Absorption was measured at 550 nm with 690 nm as reference wavelength, using a microplate reader (Synergy H1, Biotek, Bad Friedrichshall, Germany). As an additional assay for cell viability, the cell number was determined using a hemocytometer (Neubauer chamber) and trypan blue (Sigma-Aldrich).

### 2.4 HEK293 reporter gene assay

Three stably transfected human embryonic kidney HEK293 cell lines with either GPX4-specific SECIS element, SECIS-free (negative control) or 100% read-through (positive control) reporter constructs [5] were cultured in Dulbecco's Modified Eagle Medium with high glucose (DMEM; Pan-biotech, Aidenbach, Germany) with 10% (v/v) FCS, 1% (v/v) P/S, and 1% (v/v) Glutamax at 37 °C and 5% CO_2_. For reporter gene assay, 20,000 cells per well were seeded in 96-well plates, precoated with poly-l-lysine (Biochrom/Sigma-Aldrich). Cells were incubated with 0, 5, or 10 nM selenite combined with either 0, 1, or 10 μM CuSO_4_ in DMEM, containing 2.5% (v/v) FCS. As positive control, 50 μg/mL G418 was added in combination with 5 nM selenite. After 72 h of incubation, media were aspirated and 40 μL of 1x lysis buffer (Promocell, Heidenberg, Germany) were added to the wells. After a 10 min shaking step, the plates were put into a freezer to support cell lysis. Renilla luciferase activity was measured after adding 100 μL Coelenterazine (2.5 μg/mL; Promocell) to 35 μL of the cell lysates using luminescence measurement in a microplate reader (Synergy H1). Relative light units (RLU) were normalized to samples incubated with 5 nM Se only for each replicate to obtain relative read-through efficiency.

### 2.5 RNA isolation, reverse transcription, and quantitative real-time PCR

RNA of snap-frozen liver samples was isolated as previously described [29]. Briefly, total tissue RNA was isolated using Trizol Reagent (Invitrogen, ThermoFisher Scientific). Genomic DNA was eliminated with PerfeCTa DNase I (Quanta BioSciences, Beverly, MA, USA) and reverse transcription was performed using the qScript cDNA synthesis kit (Quanta BioSciences). The mRNA of HepG2 cells was isolated with the Dynabeads mRNA DIRECT kit (ThermoFisher Scientific) according to the manufacturer's description. The mRNA was reversely transcribed using the sensifast™ cDNA synthesis kit (Bioline Meridian Bioscience, Cincinnati, Ohio, USA). Real-time PCRs were performed with 1x PerfeCTa SYBR Green Supermix (Quanta, BioSciences) using cDNA-specific primers (Table 2, Eurofins Genomics, Ebersberg, Germany) at a concentration of 250 nM in a total volume of 10 μL. The Mx3005P QPCR System (Agilent Technologies, Santa Clara, CA, USA) was used with the following heating steps: 3 min at 95 °C, 40 cycles of 15 s at 95 °C, 20 s at 60 °C, and 30 s at 72 °C with all samples and standards measured in triplicates. Standard curves from diluted PCR products were used for quantification. Sample values were normalized to a composite factor based on the reference genes Hprt and Rpl13a. The quantification procedure was performed in accordance with the MIQE guidelines.

**Table 2:** Primer sequences (5‘ → 3‘).

	Gene	RefSeq-ID	Sequence
mouse	Ccs, Cu chaperone for superoxide dismutase	NM_016892.3	GATGTGATTGGCCGCAGCCT CACAGGCCAACCTCTTCCCA
	Hprt, hypoxanthine phosphoribosyltransferase 1	NM_013556.2	GCAGTCCCAGCGTCGTG GGCCTCCCATCTCCTTCAT
	Rpl13a, ribosomal protein L13a	NM_009438.5	GTTCGGCTGAAGCCTACCAG TTCCGTAACCTCAAGATCTGCT
	Mt1, metallothionein 1	NM_013602.3	CTCCTGCAAGAAGAGCTGCTGC CGCTGTTCGTCACATCAGGC
	Mt2, metallothionein 2	NM_008630.2	CTGTGCCTCCGATGGATCCT CTTGTCGGAAGCCTCTTTGCAG
human	EEFSEC, selenocysteine-specific elongation factor	NM_021937.3	CCCTAGAGAACACCAAGTTCCGAG TCAATGAGCTCTGGAATGCCCT
	GCLM, glutamate-cysteine ligase modifier subunit	NM_002061.3	GTTGACATGGCCTGTTCAGTCCT CCCAGTAAGGCTGTAAATGCTCCA
	GPX1, glutathione peroxidase 1	NM_000581.2	TACTTATCGAGAATGTGGCGTCCC TTGGCGTTCTCCTGATGCCC
	GPX2, glutathione peroxidase 2	NM_002083.4	GTGCTGATTGAGAATGTGGC AGGATGCTCGTTCTGCCCA
	GPX4, glutathione peroxidase 4	NM_002085.3	AGGCAAGACCGAAGTAAACTACAC TCTCTTCGTTACTCCCTGGCT
	HPRT, hypoxanthine phosphoribosyltransferase 1	NM_000194.2	TGGCGTCGTGATTAGTGATG GGCCTCCCATCTCCTTCAT
	MT2a, metallothionein 2a	NM_005953.3	AGGGCTGCATCTGCAAAGGG TAGCAAACGGTCACGGTCAGGG
	NQO1, NAD(P)H quinone dehydrogenase 1	NM_001025434.1	CATCACAGGTAAACTGAAGGACCC CTCTGGAATATCACAAGGTCTGCG
	PSTK, phosphoseryl-tRNA kinase	NM_153,336	TTTGAGGCCCAGTCTTGCTACC GCCCAACGAATATTTCCGAGCC
	RPL13A, ribosomal protein L13a	NM_012423.2	AGCCTACAAGAAAGTTTGCCTATCTG TAGTGGATCTTGGCTTTCTCTTTCCT
	SELENOH, selenoprotein H	NM_170746.2	GCTTCCAGTAAAGGTGAACCCGA TCAGGGAATTTGAGTTTGCGTGG
	SELENOP, selenoprotein P	NM_005410	GAAACTCCATCGCCTCATTACCAT CTGCCTATGCTGACCCTTGTG
	SELENOW, selenoprotein W	NM_003009.2	GCGGAAGTTGCAGCTACAAGTC CGGCTACCATCACTTCAAAGAACC
	SEPHS2, selenophosphate synthetase 2	NM_012,248	GACGGTTTGGGCTTCTTCAAGG TCCACAATGCCAACGATCCAC
	SEPSECS, Sep (O-phosphoserine) tRNA-Sec tRNA synthase	NM_016955.3	CTAGTGCTCCCGCTTATTCGCC CTGGACACTTGCCCTTCTCCAG
	TXNRD1, thioredoxin reductase 1	NM_015762.1	GTGTTGTGGGCTTTCACGTACTG TGTTGTGAATACCTCTGCACAGAC

### 2.6 Western blot

To prepare protein lysates, frozen cell pellets or murine tissues were homogenized in Tris buffer (100 mM Tris (Carl Roth), 300 mM KCl (Applichem, Darmstadt, Germany), pH 7.6 with 0.1% (v/v) Triton X-100 (Serva, Heidelberg, Germany), and 0.1% (v/v) protease inhibitor (Merck/Millipore, Burlington, MA, USA)) using a TissueLyser II (Qiagen, Hilden, Germany) by a 2 × 30 s homogenizing step at maximum speed. Cellular debris was removed by centrifugation (14,000 g, 10 min, 4 °C). Protein concentration was determined by Bradford analysis (Bio-Rad Laboratories, Munich, Germany). SDS polyacrylamide gel electrophoresis was followed by immunoblotting of proteins to nitrocellulose membrane. After immunoblotting membranes were gently shaken for 2 min in Ponceau-S solution (0.2% (w/v) Ponceau S (Carl Roth) with 3% (w/v) trichloroacetic acid (Carl Roth) and bands were recorded by ChemiDoc™ MP Imaging System (Bio-Rad). Subsequently, membranes were blocked in 5% (w/v) non-fat dry milk in Tris-buffered saline containing 0.1% (v/v) Tween 20 (T-TBS) for 1 h at RT. The membranes were incubated with the following primary antibodies overnight at 4 °C: rabbit *anti*-GPX1 (3120–1, epitomics, Burlingham, CA, USA, 1:5000), rabbit *anti*-GPX2 ([30], 1:5000), rabbit *anti*-GPX4 (125,066, abcam, Cambridge, UK, 1:5000), rabbit *anti*-TXNRD1 (124,954, abcam, 1:5000), rabbit *anti*-TXNRD2 (180,493, abcam, 1:1000), rabbit *anti*-SELENOH (151,023, abcam, 1:500 (mouse tissue), 1:1000 (cell culture)), rabbit *anti*-MT (192,385, abcam, 1:1000), rabbit *anti*-CCS (137,131, abcam, 1:5000), rabbit *anti*-NQO1 (34,173, abcam, 1:4000), and rabbit *anti*-SELENOW (600-401-A29, Rockland, Gilbertsville, PA, USA, 1:1000). As secondary antibody horseradish peroxidase-conjugated goat anti-rabbit IgG (1:50,000, 7074S, Cell Signaling, Danvers, MA, USA) was incubated for 1 h in 5% (w/v) non-fat dry milk in T-TBS at RT. Proteins were detected using SuperSignal™ West Dura (ThermoFisher Scientific) and band intensities were quantified densitometrically by the ChemiDoc™ MP Imaging System (Bio-Rad). Protein expression was normalized to ponceau staining.

### 2.7 Enzyme activities

The protein lysates (see section ‘Western blot’) were used to measure total activity of GPX [31], TXNRD [32], and NAD(P)H quinone dehydrogenase 1 (NQO1) [33] as described previously. Briefly, GPX activity was measured using a NADPH-consuming glutathione reductase (GR)-coupled assay, and TXNRD activity was determined by NADPH-dependent reduction of 5,5′-dithio-bis-(2-nitrobenzoic acid) (DTNB) to 2-nitro-5-thiobenzoic acid (TNB). NQO1 activity was conducted using the menadione-mediated reduction of MTT. For measurement of direct effects on the GPX and TXNRD assay both containing EDTA in the reaction mix, Cu, BCS, and TTM were added in increasing concentrations 15 min prior to measuring of enzyme activities to the cell lysates obtained from cells cultured with 50 nM selenite for 72 h. All enzymatic activity measurements were conducted in triplicates using a 96-well plate and a microplate reader (Synergy H1) and were normalized to protein content (Bradford analysis, see section ‘Western blot’).

### 2.8 Determination of free thiols and total GSH

Measurement of free thiols and GSH was conducted as described earlier [34]. Briefly, supernatants of cultured cells were used to determine free thiols by thiol-mediated reduction of DTNB to TNB. TNB was measured photometrically at 412 nm and normalized to the protein content of the obtained cell lysates. For total GSH determination, cell pellets were lysed in 10 mM HCl (Carl Roth) using ultrasonification (10x, 80% amplitude, 0.5 s) followed by centrifugation (8000×*g*, 30 s, RT) to remove cellular debris. Supernatants were incubated for 10 min with 5% (w/v) 5-sulfosalicylic acid (Sigma-Aldrich) at RT to precipitate proteins. After an additional centrifugation step (8000×*g*, 15 min, 4 °C), samples were used to measure total GSH. The GR-mediated NADPH-consuming reduction of GSSG was coupled to the formation of TNB, which was measured photometrically at 412 nm. The total GSH content was calculated using a standard curve and was normalized to the protein content of samples. For GSH depletion, cells were treated for 24 h with 0.25 mM buthionine-sulfoximine (BSO, Sigma-Aldrich).

### 2.9 Measurement of Se and Cu content

Cu content of cell lysates and media samples was measured using a bench-top total reflection X-ray fluorescence (TXRF) spectrometer (S2 Picofox™, Bruker Nano GmbH, Berlin, Germany). As internal standard 1 mg/mL Yttrium (Merck/Millipore) was used. 10 μL of each sample were placed on siliconized quartz glass carriers and dried at 40 °C. Samples were measured in duplicates for up to 500 s. Cu and Se content in liver and colon tissue and Se content of HepG2 cells were determined using ICP-MS/MS. Preparation of samples was described previously [29]. Briefly, samples were weighted into PTFE microwave vessels. HNO_3_ (65% (v/v), Suprapure®, Merck/Millipore), H_2_O_2_ (30% (v/v), Sigma-Aldrich/Merck), rhodium (Rh) as internal standard, and ^77^Se as isotope dilution standard were added before digestion using a Mars 6 microwave digestion system (CEM, Kamp-Lintfort, Germany). After digestion, samples were diluted to achieve final concentrations of 2.93% (v/v) HNO_3_, 10 μg/L^77^Se, and 1 μg/L Rh. The samples were measured using ICP-MS/MS (8800 ICP-QQQ-MS, Agilent Technologies) and analyzed as described earlier [29]. Certified reference materials, namely fish muscle (ERM BB-422) and pig kidney (ERM BB-186) were used as quality control of digestion and to cross validate TE analysis using TXRF and ICP-MS/MS.

### 2.10 Statistics

Data are given as mean + SD. Statistical significance was calculated using GraphPad Prism version 8 (San Diego, CA, USA) with one-way or two-way analysis of variance (ANOVA) and Bonferroni's post-test as indicated in the figure legends. Correlation analysis was performed using calculation of Pearson correlations coefficients. A p-value below 0.05 was considered statistically significant.

## 3 Results

### 3.1 Cu inhibits the mRNA expression of GPX1 and selenoprotein W but does not modulate the cellular redox homeostasis

**Fig. 1:**
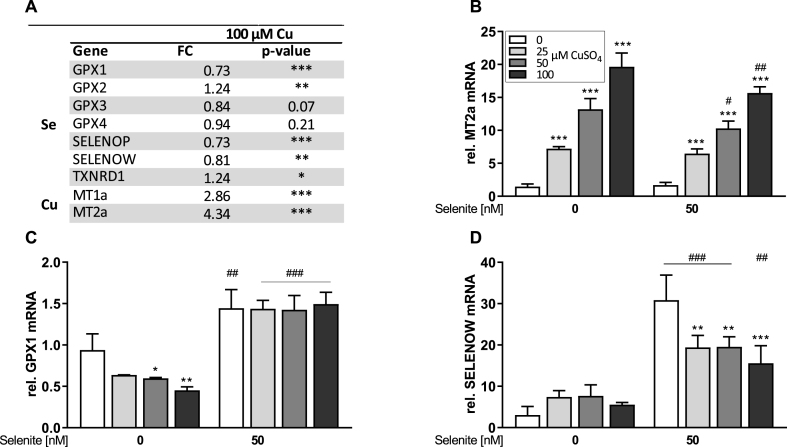
**Expression of Se- and Cu-dependent genes in HepG2 cells**. Microarray data provided by GEO Profiles (GEO Series Accession No. GSE9539) [35] obtained from HepG2 cells treated for 24 h with 100 μM CuSO_4_ (A). Data are given as fold change (FC) relative to the untreated control (n = 3). qPCR results of various Se- and Cu-responsive genes analyzed in HepG2 cells cultured with increasing Cu concentrations (0, 25, 50 or 100 μM) combined with or without 50 nM selenite for 48 h (B–D). Gene expression was normalized to the reference genes RPL13A and HPRT. Untreated cells of the first replicate were set as 1. Data are depicted as mean + SD (n = 3). Statistical analyses were based on two-way ANOVA with Bonferroni's post-test. *p < 0.05; **p < 0.01; ***p < 0.001 vs. 0 μM CuSO_4_ and ^#^p < 0.05; ^##^p < 0.01; ^###^p < 0.001 vs. 0 nM Se.

We used data provided via GEO profiles from a microarray study performed in HepG2 cells treated with 100 μM CuSO_4_ [35]. Searching for selenoprotein transcripts within the whole transcriptome, data revealed that out of the 25 human genes encoding for selenoproteins, seven were detected by this microarray approach. While GPX3 and GPX4 were not significantly modulated by Cu treatment, five transcripts were significantly altered. Out of those, GPX2 and TXNRD1 mRNA levels were upregulated and GPX1, SELENOP, and SELENOW mRNA levels were downregulated. As expected, high fold changes were observed for the two Cu-responsive genes metallothionein MT1A and MT2A (Fig. 1A).

**Fig. S1:**
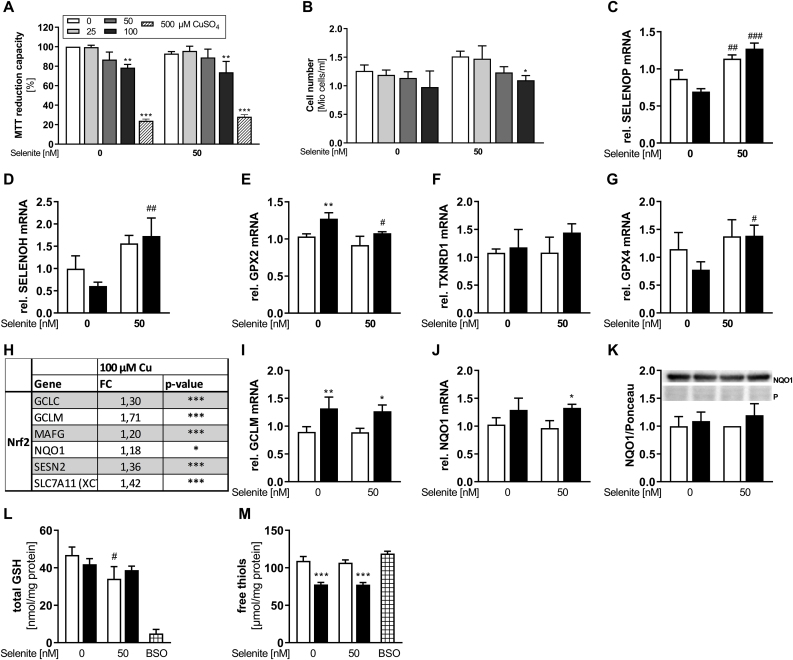
**Markers of cytotoxicity and redox status**. MTT reduction capacity (A) and cell number (B) of HepG2 cells cultured with increasing Cu concentrations (0, 25, 50 or 100 μM) in combination with or without 50 nM selenite for 72 h. 500 μM Cu was used as positive control for MTT reduction capacity and untreated cells were set as 100%. HepG2 cells were treated with Cu (0 or 100 μM) in combination with or without 50 nM selenite for 48 h (C-G, I, J) or 72 h (K-M). Gene expression of selenoproteins and Nrf2 target genes and protein expression of NQO1 were analyzed using qPCR or Western Blot (C–K). Gene expression was normalized to the reference genes RPL13A and HPRT. Untreated cells of the first replicate were set as 1. Protein expression was normalized to Ponceau staining (P). Microarray data provided by GEO Profiles (GEO Series Accession No. GSE9539) [35] obtained from HepG2 cells treated for 24 h with 100 μM CuSO_4_ (C). Data are given as fold change (FC) relative to the untreated control (n = 3). Total GSH (G) and free thiols (H) were measured photometrically in cells or supernatant, respectively and were normalized to protein content of the cell lysate. 24 h of BSO treatment was used as positive control. Data are depicted as mean + SD (n = 3-4). *p < 0.05; **p < 0.01; ***p < 0.001 vs. 0 μM CuSO_4_ and ^#^p < 0.05; ^##^p < 0.01; ^###^p < 0.001 vs. 0 nM Se calculated based on two-way ANOVA with Bonferroni's post-test.

As selenoprotein mRNA levels are also affected by Se, we extended the microarray experiment by adding lower concentrations of CuSO_4_ (25, 50, and 100 μM) combined with a low or adequate Se supply (0 and 50 nM selenite). Cell number and metabolic activity (MTT reduction activity) as measures for cell viability were unaffected by the two lower doses of CuSO_4_ while treatment with 100 μM CuSO_4_ resulted in a reduction of about 20% for both parameters independent of the Se status of the cells (Figs. S1A and B). Using qPCR, we observed a concentration-dependent upregulation of MT2a mRNA by CuSO_4_ up to a fold change of 20 which was decreased by co-treatment with selenite (Fig. 1B). mRNA levels of GPX1 (Fig. 1C) and SELENOW (Fig. 1D) were significantly downregulated by Cu, but this reduction was only detectable either under -Se conditions for GPX1 or under +Se conditions for SELENOW. In addition, mRNA expression levels of SELENOP (Fig. S1C) and SELENOH (Fig. S1D) showed a trend for a Cu-induced downregulation under -Se conditions. GPX2 mRNA levels were upregulated by Cu (Fig. S1E) while TXNRD1 (Fig. S1F) and GPX4 (Fig. S1G) mRNA levels were unaffected by Cu.

As GPX2 and TXNRD1 are regulated by Nrf2, we next aimed to characterize effects of the experimental set-up on the cellular redox homeostasis. Based on the microarray data, additional Nrf2 target genes were upregulated by Cu including both subunits of the glutamate-cysteine ligase (GCL; Fig. S1H), which could be confirmed by qPCR for GCLM (Fig. S1I). In addition, the Nrf2 target gene NQO1 was significantly induced in the microarray and qPCR (Fig. S1J). However, this effect could not be observed for NQO1 protein expression (Fig. S1K). The intracellular GSH concentration was not affected by Cu but slightly decreased in selenite-treated cells (Fig. S1L), while the concentration of extracellular free thiols was downregulated by Cu independent of the Se supply (Fig. 1M). This indicates that moderately increasing the Cu supply above normal levels does not result in oxidative stress but induces a very mild Nrf2 response.

### 3.2 Cu modulates protein expression of glutathione peroxidases

**Fig. 2:**
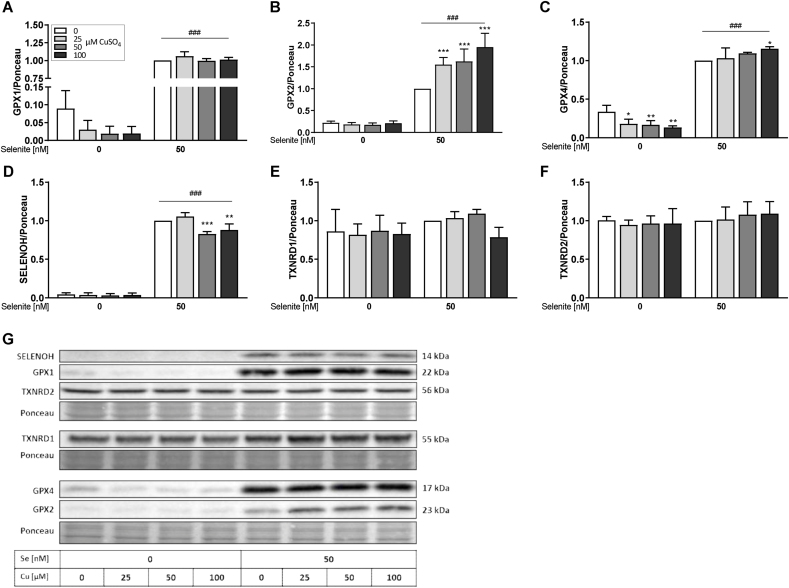
**Cu modulates the expression of several selenoproteins.** HepG2 cells were treated with increasing Cu concentrations (0, 25, 50 or 100 μM) in combination with or without 50 nM selenite for 72 h. Protein expression was determined using Western blot, normalized to Ponceau staining. Samples with Se treatment and without Cu were set as 1 (A–F). Representative blots are shown (G). Data are depicted as mean + SD (n = 3-4). Statistical analyses were based on two-way ANOVA with Bonferroni's post-test. *p < 0.05; **p < 0.01; ***p < 0.001 vs. 0 μM CuSO_4_ and ^###^p < 0.001 vs. 0 nM Se.

**Fig. S2:**
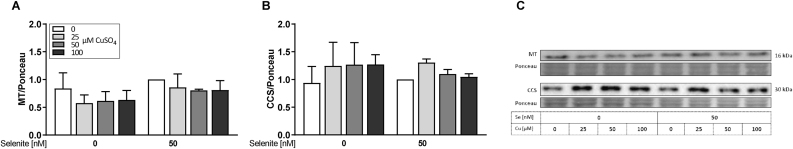
**Cu responsive proteins are unaffected by the Cu and Se treatment.** HepG2 cells were treated with increasing Cu concentrations (0, 25, 50 or 100 μM) in combination with or without 50 nM selenite for 72 h. Protein expression was determined using Western blot, normalized to Ponceau staining. Samples with Se treatment and without Cu were set as 1 (A, B). Representative blots are shown (C). Data are depicted as mean + SD (n = 3-4). Statistical analyses were based on two-way ANOVA with Bonferroni's post-test.

Next, we aimed to analyze if the combined Se and Cu treatments not only affect mRNA expression but also protein levels. As expected, protein expression of GPX1, GPX2, GPX4, and SELENOH (Fig. 2A–D) increased in a Se-dependent manner while TXNRD1 and TXNRD2 (Fig. 2E and F) were unaffected by the Se status. The most pronounced inhibitory Cu effects were observed for GPX4 which was decreased by Cu treatment under -Se conditions but rather unaffected under +Se conditions (Fig. 2C). A comparable effect was observed for GPX1 which however only showed a trend for a decreased expression in the 100 μM Cu treatment group without Se (p = 0.08) (Fig. 2A). In contrast, GPX2 protein expression was unaffected by Cu under -Se conditions but increased with Cu treatment under +Se conditions (Fig. 2B). SELENOH, TXNRD1, and TXNRD2 (Fig. 2D–F) protein expression levels were rather unaffected by Cu treatment. The expression of the Cu marker proteins MT and CCS was not modulated by any of the treatment conditions (Figs. S2A–C).

### 3.3 GPX and TXNRD activities are downregulated by Cu treatment

**Fig. 3:**
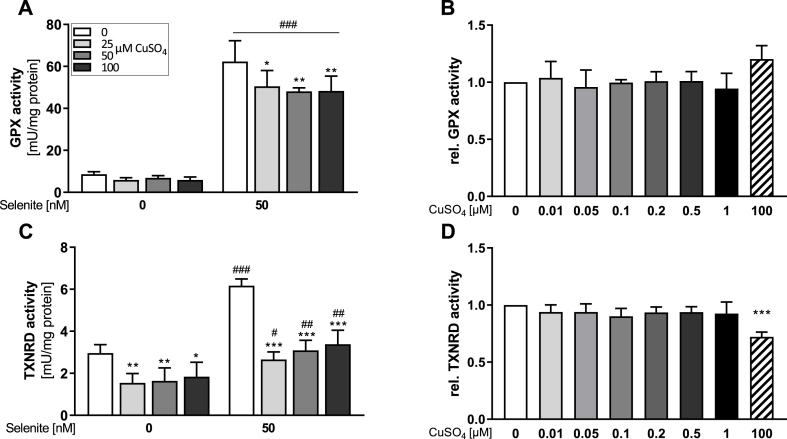
**Cu decreases selenoprotein activity, but does not directly affect enzyme activity within the assay.** HepG2 cells were cultured with increasing Cu concentrations (0, 25, 50 or 100 μM) in combination with or without 50 nM selenite for 72 h (A, C). Lysates of selenite supplemented (50 nM for 72 h) cells were used to measure the direct impact of Cu on enzyme activities (B, D). Increasing concentrations of Cu were added 15 min prior to measurement of enzyme activities and were normalized to lysates without additional Cu. Activities of GPX (A, B) and TXNRD (C, D) were measured photometrically and normalized to protein content. Data are depicted as mean + SD (n = 3-4). Statistical analyses were based on two-way ANOVA with Bonferroni's post-test (A, C) or one-way ANOVA (B, D) with Bonferroni's post-test. *p < 0.05; **p < 0.01; ***p < 0.001 vs. 0 nM CuSO_4_ and ^#^p < 0.05; ^##^p < 0.01; ^###^p < 0.001 vs. 0 nM Se.

**Fig. S3:**
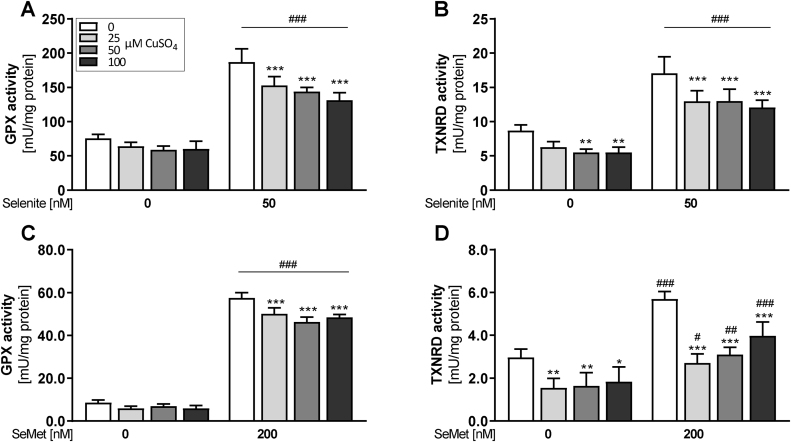
**Cu decreases selenoprotein activity in HT29 cells and independent of the selenocompound.** Measurement of GPX and TXNRD enzyme activity in HT29 (A, B) and HepG2 cells (C, D) treated with increasing Cu concentrations (0, 25, 50 or 100 μM) in combination with or without 50 nM selenite (A, B) or 200 nM selenomethionine (C, D) for 72 h, respectively. Data are depicted as mean + SD (n = 3-4). *p < 0.05; **p < 0.01; ***p < 0.001 vs. 0 μM CuSO_4_ and ^#^p < 0.05; ^##^p < 0.01; ^###^p < 0.001 vs. 0 nM Se calculated based on two-way ANOVA with Bonferroni's post-test.

Next, we aimed to identify potential Cu effects on total enzyme activities of GPX and TXNRD. Both GPX and TXNRD activities were upregulated by an increasing Se supply (Fig. 3A, C). Cu treatment resulted in a significant decrease of total GPX activity down to about 80% which was, however, only detectable in selenite-treated cells (Fig. 3A). In contrast, TXNRD activity was inhibited by Cu to about 50% which was independent of the cellular Se status (Fig. 3C). These Cu-induced effects on GPX and TXNRD activities were confirmed using another cell line, namely HT-29 (Figs. S3A and B). In addition, we used SeMet as an alternative selenocompound for studying interactions between Cu and Se. Also in SeMet-treated cells, Cu co-treatment efficiently inhibited GPX and TXNRD activities (Figs. S3C and D). To exclude that Cu directly interfered with the assays, e.g. by binding to NADPH, increasing Cu concentrations were added to the reaction mixture of the GPX (Fig. 3B) or TXNRD (Fig. 3D) assay 15 min prior to measurement. None of the tested Cu concentrations affected the GPX assay (Fig. 3B). TXNRD activity was stable up to 1 μM of added Cu but inhibited by a very high CuSO_4_ concentration of 100 μM (Fig. 3D). But even this high Cu concentration was not resulting in a comparable inhibition of TXNRD activity as observed in cultured cells (Fig. 3C).

### 3.4 Cu treatment decreases read-through and thus UGA recoding efficiency

**Fig. 4:**
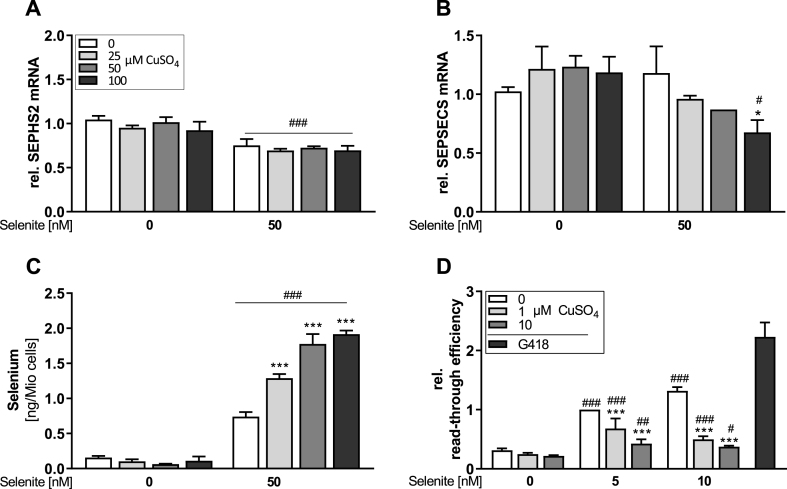
**Cu affects gene expression of the selenoprotein synthesis machinery, the cellular Se content and read-through efficiency.** HepG2 cells were cultured with increasing Cu concentrations (0, 25, 50 or 100 μM) in combination with or without 50 nM selenite for 48 h. Gene expression was analyzed by qPCR and normalized to the reference genes RPL13A and HPRT (A, B) and untreated cells of first replicate were set as 1. The Se content of cell lysates was measured using ICP-MS/MS (C). Read-through efficiency was measured using HEK293 cells stably transfected with a reporter gene vector containing the SECIS element of GPX4. Cells were cultured with 1 or 10 μM CuSO_4_ in combination without or with 5 and 10 nM selenite for 72 h. Read-through efficiency was determined by luminescence measurement and was shown relative to cells treated with 5 nM selenite (D). G418 (+5 nM Se) was used as positive control. Data are depicted as mean + SD (n = 3). Statistical analyses were based on two-way ANOVA with Bonferroni's post-test. *p < 0.05; ***p < 0.001 vs. 0 μM CuSO_4_ and ^#^p < 0.05; ^##^p < 0.01; ^###^p < 0.001 vs. 0 nM Se.

**Fig. S4:**
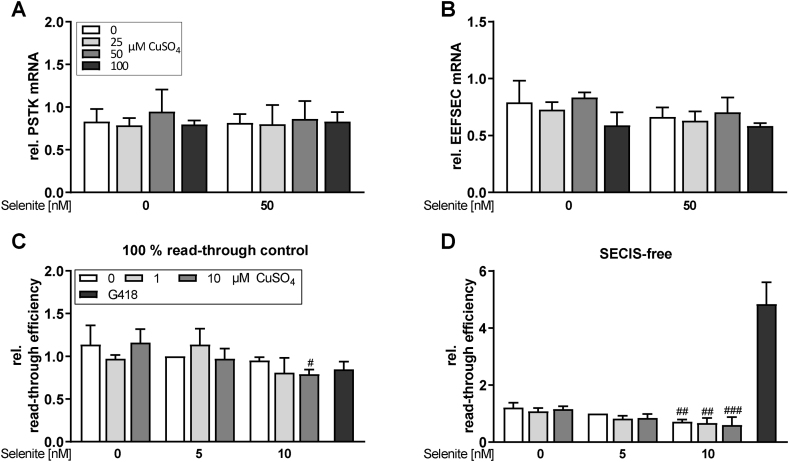
**Read-through efficiency of control reporters.** HepG2 cells were cultured with increasing Cu concentrations (0, 25, 50 or 100 μM) in combination with or without 50 nM selenite for 48 h. Gene expression was analyzed by qPCR and normalized to the reference genes RPL13A and HPRT (A, B) and untreated cells of first replicate were set as 1. Positive (C) and negative (D) reporter read-through efficiency was measured using HEK293 cells stably transfected with a UGC triplet (= 100% read-through) or a SECIS-free sequence, respectively. Cells were cultured with 1 or 10 μM CuSO_4_ in combination without or with 5 and 10 nM selenite for 72 h. Read-through efficiency was determined using luminescence measurement and normalized to cells treated only with 5 nM selenite. G418 (+5 nM Se) was used as positive control. Data are depicted as mean + SD (n = 3). ^#^p < 0.05; ^##^p < 0.01; ^###^p < 0.001 vs. 0 nM Se calculated based on two-way ANOVA with Bonferroni's post-test.

To clarify whether Cu affects the selenoprotein synthesis machinery, mRNA expression levels of genes encoding for factors essential for selenoprotein synthesis were analyzed. Out of the four tested genes, only SEPHS2 was sensitive towards the Se status and was downregulated under conditions of Se supply (Fig. 4A). SEPSECS expression was diminished upon treatment with 100 μM CuSO_4_ which was only observed under +Se conditions (Fig. 4B). PSTK and EEFSEC expression was neither affected by Se nor by Cu treatment (Figs. S4A and B). To verify intracellular Se availability for cells upon Cu treatment, the cellular Se content was determined. The Se content was increased with increasing Cu concentrations and was almost doubled with highest Cu concentration under +Se conditions. Under -Se conditions, there was no Cu effect on intracellular Se levels (Fig. 4C). Another way of modulating selenoprotein expression is via affecting the SECIS read-through efficiency. We used the SECIS element of GPX4 to test for a potential Cu effect. Cu downregulated the read-through efficiency in a concentration-dependent manner under +Se conditions. G418 was used as a positive control and doubled read-through efficiency (Fig. 4F). Both, control cells transfected with the positive control vector with 100% read-through and the SECIS-free negative control vector were unaffected by Cu treatment (Figs. S4C and D).

### 3.5 Reversal of Cu effects on selenoprotein expression and activity by Cu chelators

**Fig. 5:**
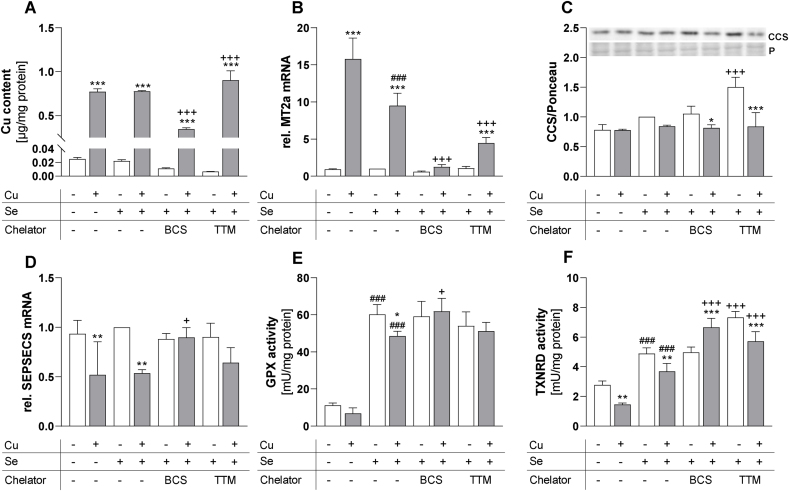
**Cu-induced effects could be reversed by co-treatment with the Cu chelators BCS and TTM.** HepG2 cells were treated with 100 μM CuSO_4_ in combination with or without 50 nM selenite for 72 h. After 48 h of incubation, the two chelators bathocuproine disulfonic acid (BCS, 400 μM) or tetrathiomolybdate (TTM, 75 μM) were added to the cells. Cu content (A) was measured using TXRF and normalized to protein content. Gene expression (B, D) was determined via qPCR and normalized to the reference genes RPL13A and HPRT. Protein expression (C) was normalized to Ponceau staining (P). Cells with Se, but without chelator or Cu treatment were set as 1. Enzyme activities of GPX and TXNRD (E, F) were measured photometrically. Data are depicted as mean + SD (n = 4). Statistical analyses were based on two-way ANOVA with Bonferroni's post-test. *p < 0.05; **p < 0.01; ***p < 0.001 vs. 0 μM CuSO_4_; ^###^p < 0.001 vs. 0 nM Se, and ^+^p < 0.05; ^+++^p < 0.001 vs. -chelator.

**Fig. S5:**
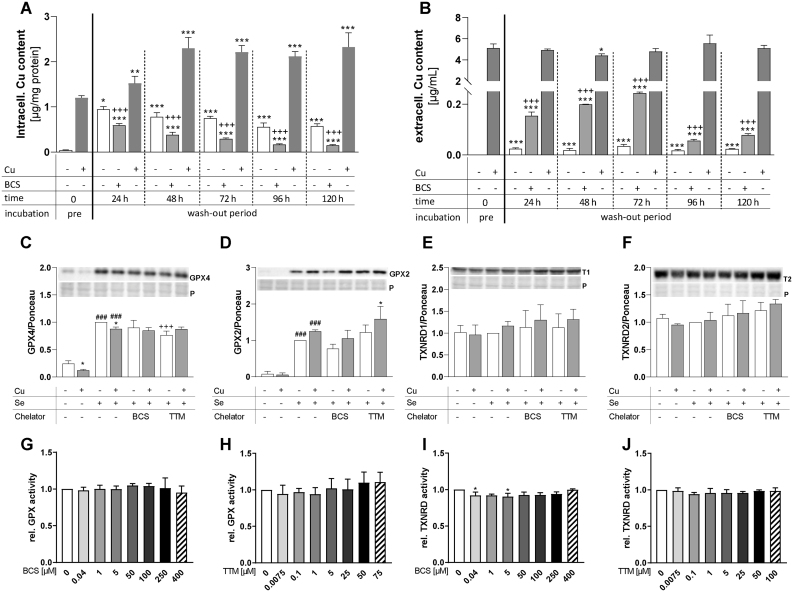
**The Cu chelator BCS affects Cu content, but showed no impact on selenoprotein activity and expression.** HepG2 cells were cultured with or without 100 μM CuSO_4_ for 72 h. After this period, all samples with prior CuSO_4_ treatment were divided into three cultivation groups (-Cu/-BCS, -Cu/+BCS, +Cu/-BCS) for another five days with media exchange after three days. Every day one sample of each group was harvested and Cu content of cells (A) and media (B) was determined using TXRF. *p < 0.05; **p < 0.01; ***p < 0.001 vs. 72 h + CuSO_4_; ^+++^p < 0.001 vs. 0 μM CuSO_4_ at the same time point, based on two-way ANOVA with Bonferroni's post-test excluding the +Cu/-BCS group for statistical analysis. HepG2 cells were cultivated with 100 μM CuSO_4_ in combination with or without 50 nM selenite for 72 h. After 48 h of incubation, the two chelators bathocuproine disulfonic acid (BCS, 400 μM) or tetrathiomolybdate (TTM, 75 μM) were added to the cells. Protein expression (T1 = TXNRD1; T2 = TXNRD2) was determined using Western blot and normalized to Ponceau staining (P). Samples with Se but without further treatment were set as 1 (C–F). *p < 0.05 vs. 0 μM CuSO_4_; ^###^p < 0.001 vs. 0 nM Se, and ^+++^p < 0.001 vs. -chelator calculated based on two-way ANOVA with Bonferroni's post-test. Lysates of selenite supplemented cells (50 nM for 72 h) were used to measure the direct impact of BCS or TTM on enzyme activities. Increasing concentrations of BCS or TTM were added 15 min prior to measurement of enzyme activities and were normalized to lysates without additional BCS or TTM incubation, respectively. Activities of GPX (G, H) and TXNRD (I, J) were measured photometrically. Data are depicted as mean + SD (n = 3-4). *p < 0.05 vs. 0 μM BCS or TTM calculated based on one-way ANOVA with Bonferroni's post-test.

To study the Cu specificity of the effects observed, we established treatment conditions with two different Cu specific chelators, namely BCS and TTM. The Cu content of the cells increased by Cu treatment but remained unaffected by Se co-treatment (Fig. 5A). After 24 h of BCS treatment, the intracellular Cu content decreased to 55%, whereas intracellular Cu increased in response to TTM treatment (Fig. 5A). These findings are supported by previously published data showing that BCS is an extracellular chelator [36]. BCS efficiently decreased the intracellular Cu content not only after 24 h of treatment (Fig. 5A), but also over a period of five days, when supplied to Cu-supplemented cells (Fig. S5A). BCS was able to sequester Cu from cells which resulted in Cu accumulation in the media (Fig. S5B). In contrast, TTM is known to be taken up into cells and is supposed to bind and accumulate Cu there [36] which results in higher cellular Cu levels (Fig. 5A). However, this TTM-bound Cu is not available as free Cu and is thus less bioactive.

Accordingly, co-treatments with each of the two chelators were used to test whether Cu-induced effects on selenoproteins and Cu-related biomarkers can be reversed. The Cu-induced increase of MT2a mRNA expression was efficiently diminished by the two chelators, but most strongly by BCS reaching almost basal MT2a expression levels (Fig. 5B). Again, MT2a expression was also repressed by an increasing Se supply. CCS protein expression was increased when TTM but not BCS was added to the -Cu groups (Fig. 5C). The Cu-induced downregulation of SEPSECS was reversed by BCS, but not by TTM (Fig. 5D). Co-treatment with each of the two chelators blocked the Cu-induced inhibition of GPX activity (Fig. 5E). The protein expression of different GPXs again was only marginally affected (Figs. S5C and D). We also measured a putative direct influence of BCS and TTM on the GPX activity assay, which was not observed (Figs. S5G and H). The Cu-induced inhibition of TXNRD activity was not only reversed by BCS treatment, but TXNRD activity even further increased above basal levels. In contrast, TTM did not reverse the Cu-mediated inhibition of TXNRD activity. Interestingly, TXNRD activity was generally increased in TTM-treated cells (Fig. 5F). Although effects on TXNRD activity were detectable, the protein expression of neither TXNRD1 nor TXNRD2 was affected by Cu or the chelators (Figs. S5E and F). As shown for GPX activity, the two chelators had no direct effect on the TXNRD activity assay (Figs. S5I and J).

### 3.6 *In vivo* interactions of Se and Cu

**Fig. 6:**
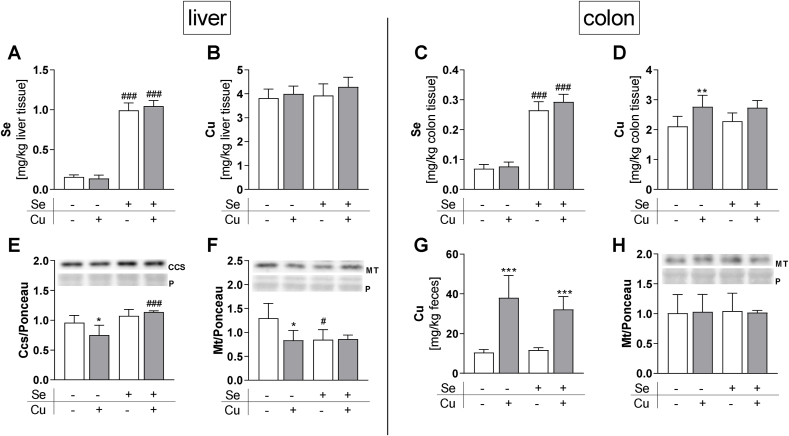
**Dietary intervention with the TEs Cu and Se.** Se and Cu contents in liver (A, B), and colon (C, D) of mice supplied with suboptimal (0.02/1.6 ppm) or adequate (0.15/6 ppm) amounts of Se and Cu were determined using ICP-MS/MS. Protein expression (E, F, H) was normalized to Ponceau staining (P). Data are depicted as mean + SD (n = 5). *p < 0.05; ***p < 0.001 vs. -Cu; ^#^p < 0.05; ^##^p < 0.01; ^###^p < 0.001 vs. -Se calculated based on two-way ANOVA with Bonferroni's post-test.

**Fig. S6:**
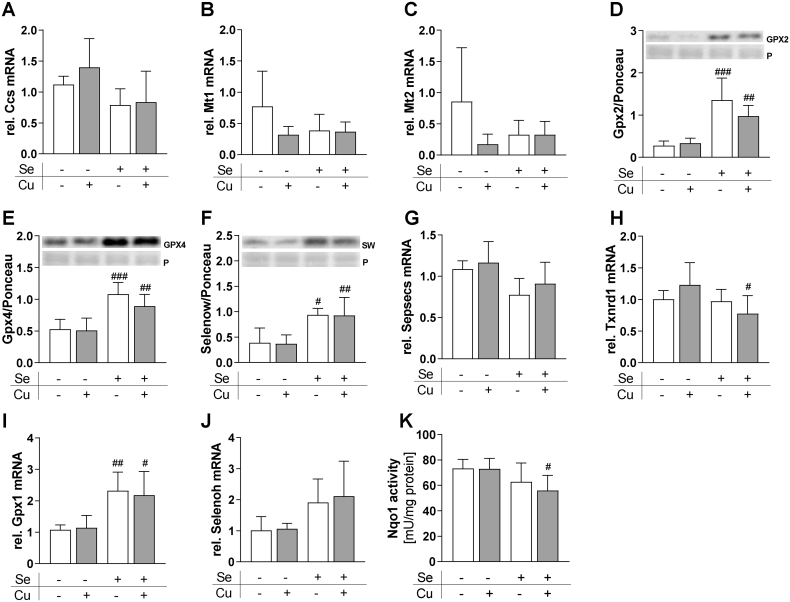
**Hepatic gene and protein expression upon dietary intervention with the TEs Cu and Se.** Hepatic gene and protein expression of mice supplied with suboptimal (0.02/1.6 ppm) or adequate (0.15/6 ppm) amounts of Se and Cu were determined using qPCR and Western blot, respectively. Gene expression was normalized to the reference genes Rpl13a and Hprt (A-C, G-J). Protein expression (SW = Selenow) was normalized to Ponceau staining (P). Nqo1 activity was measured photometrically and normalized to protein content. Data are depicted as mean + SD (n = 5). ^#^p < 0.05; ^##^p < 0.01; ^###^p < 0.001 vs. -Se calculated based on two-way ANOVA with Bonferroni's post-test.

To further elucidate if Cu interferes with selenoprotein synthesis and activity also *in vivo*, we performed a mouse study with suboptimal or adequate amounts of Se and Cu supplied by the drinking water. The Se and Cu status of all mice was characterized by measuring the concentrations of both TEs in liver samples as the central metabolic organ for TEs and in colon samples to study local effects between the luminal content and the organism. The Se concentrations of both liver (Fig. 6A) and colon (Fig. 6C) were strongly different in relation to the Se supply, indicating that the feeding conditions were well suitable to modulate the Se status. However, even though there was a 3.75-fold difference in Cu supply between -Cu and +Cu mice, no differences in hepatic Cu levels were observed (Fig. 6B). A difference in Cu concentrations was detectable in the colon only (Fig. 6D), which was even far more pronounced in the feces (Fig. 6G). To further characterize the Cu status of the mice, expression levels of Cu-dependent proteins were analyzed. Hepatic Ccs protein expression was downregulated by Cu which was, however, only detectable under -Se and not under +Se conditions (Fig. 6E). Ccs transcript levels were not significantly modulated by Cu or Se in the liver (Fig. S6A). Mt protein expression was upregulated under conditions of low Se and Cu supply in the liver (Fig. 6F), but not in the colon (Fig. 6H). The mRNA expression in the liver of both, Mt1 and Mt2 was not significantly affected by Se or Cu (Figs. S6B and C) but revealed a comparable pattern as shown on protein level with higher expression under -Cu/-Se conditions. Based on this, we concluded that the Se status was successfully modulated systemically while the Cu status was only locally modulated in the colon and not in the liver.

**Fig. 7:**
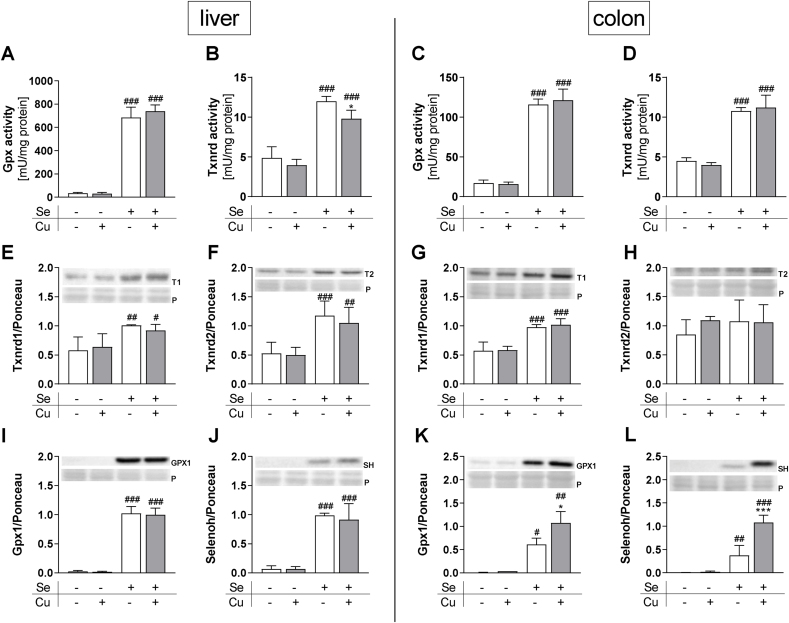
**Activity and expression of selenoproteins in vivo.** Enzyme activities of GPX (A, C) and TXNRD (B, D) and selenoprotein expression in liver and colon (E–L) of mice supplied with suboptimal (0.02/1.6 ppm) or adequate (0.15/6 ppm) amounts of Se and Cu were determined photometrically or using Western blot, respectively. Proteins (T1 = Txnrd1; T2 = Txnrd2; SH = Selenoh) were normalized to Ponceau staining (P). Data are depicted as mean + SD (n = 5). *p < 0.05; ***p < 0.001 vs. -Cu; ^#^p < 0.05; ^##^p < 0.01; ^###^p < 0.001 vs. -Se calculated based on two-way ANOVA with Bonferroni's post-test.

Next, we studied whether the Cu-induced effects on selenoproteins can be detected *in vivo* despite the marginal changes in systemic Cu status. While hepatic Gpx activity was not modulated by Cu (Fig. 7A), hepatic Txnrd activity was repressed as already observed *in vitro*, however to a smaller extent and only under +Se conditions (Fig. 7B). Hepatic selenoprotein expression was not affected by Cu (Fig. 7E, F, I, J, S6D-F). In the colon, neither Gpx (Fig. 7C) nor Txnrd (Fig. 7D) activity was downregulated by Cu, which was in line with stable Txnrd1 and Txnrd2 protein expression (Fig. 7G and H). In contrast to the *in vitro* results, colonic protein expression of Gpx1 was not downregulated but even upregulated by Cu under +Se conditions (Fig. 7K). Also, Selenoh showed a Cu-induced upregulation of protein expression in colon tissue (Fig. 7L). Hepatic Nqo1 activity was not affected by the Cu supply (Fig. S6K).

## 4 Discussion

Metabolism of the single TEs, Se and Cu, is characterized well, but interactions of both are rarely investigated. Therefore, we addressed the question of whether Cu interferes with Se metabolism in *in vitro* and *in vivo* experiments. We performed a mouse feeding study with suboptimal (0.02/1.6 ppm) and adequate (0.15/6 ppm) amounts of Se and Cu, respectively, which were supplied via the drinking water. This way, we aimed to address dietary changes of these two TEs in a physiologically relevant concentration range. For Se, both the hepatic concentration (Fig. 6A) and total Gpx activity (Fig. 7A) were downregulated to 14% or 4% in relation to the +Se group, which is in line with previous feeding experiments using the Se-deficient torula yeast diet [37,38]. However, the Cu status was affected only marginally by our intervention, because the low Cu content of the diet was obviously enough to maintain Cu homeostasis efficiently. Hepatic Cu concentrations were unaffected (Fig. 6B) which has previously been described with an even lower Cu supply [39]. Hepatic Cu only responds to severe feeding-induced Cu deficiencies [19,40,41] or in knockout mouse models e.g., for Ctr [42,43] or Atp7b [44]. Even though, there was no effect on Cu concentrations, Cu-responsive proteins such as Mt and Ccs were upregulated in the -Se/-Cu group (Fig. 6E and F). In contrast to the liver, the Cu concentration was decreased by the low Cu diet in the colon (Fig. 6D) but the effect was rather small. In response to low dietary intake, the intestinal Cu absorption strongly increases in mice [39] and in humans [45], leading to very low fecal content compared to adequately Cu-supplied mice [39]. Also herein, the treatment effect on the fecal Cu concentration was most pronounced (Fig. 6G). Overall, we successfully modulated the Se status whereas the Cu status remained largely unaffected by our experimental design.

The following results were obtained regarding *in vivo* interactions of both elements: (1) no effect on Cu levels in colon and liver after modulating the Se status from an adequate towards a suboptimal supply. This is in line with previous studies showing stable hepatic Cu levels with increasing Se concentrations [46,47]. However, we observed Cu-induced effects on Cu-responsive proteins such as Mt and Ccs (Fig. 6E and F) that were more pronounced under Se deficiency compared to an adequate Se supply indicating an interplay which warrants further investigation. (2) *Vice versa*, we did not observe any effect of Cu on the Se concentration in the liver or colon, but this might be due to the fact that the Cu status was only very marginally affected by our dietary intervention. Unexpectedly, we observed an upregulation of Gpx1 and Selenoh in the colon of the +Se/+Cu group (Fig. 7K and L). Atp7b knockout mice with hepatic Cu accumulation have increased levels of Selenoh in liver nuclei. The nuclear abundance of Selenoh in these mice is supposed to be connected to oxidative stress as a result of excessive Cu accumulation [48]. However, this is unlikely to be the case in our +Se/+Cu mice, as the activity of the Nrf2 target gene Nqo1 as indicator for the hepatic redox balance was not increased but rather decreased in mice of this group (Fig. S6K). In sheep, an increase in hepatic Se concentrations was observed following Cu administration [49] which was also observed herein in HepG2 cells (Fig. 4C).

Thus, under low to adequate conditions, there are only modest interactions of Se and Cu, however, when considering adequate to supplemented Cu concentrations, as we did in our *in vitro* experimental setting, we observed that Cu substantially interferes with selenoprotein synthesis at different levels. First, there was a Cu-dependent downregulation of transcript levels of GPX1, and SELENOW which are known to be sensitive towards a limited Se supply [50,51]. Cu even enhanced the decrease of GPX1 under low Se conditions (Fig. 1C), thus, worsening functional consequences of a Se deficiency. Interestingly, mRNA levels of SELENOW were only downregulated by Cu under conditions of an adequate Se supply (Fig. 1D) indicating that in case of SELENOW obviously higher Se concentrations are needed to upregulate mRNA levels when Cu levels are high. So far, Cu effects have been attributed mainly to an increase in oxidative stress upon Cu treatment. However, this is not likely to be the case in our experimental setting. If the cellular redox homeostasis is of relevance here, one would expect an upregulation of selenoprotein transcripts by Cu instead of a downregulation [9], which we also observed for Nrf2 target genes (Figs. S1E and F, H–K). In previous experiments, incubation of HepG2 or neuroblastoma cells with 200 μM Cu decreased p53 reporter activity and mRNA expression of GPX1 [52,53], which is known to be regulated via p53 [54,55]. However, the inhibition of p53 activity by Cu was observed with 200 μM only and not with lower Cu concentrations [52] which we used herein. SELENOH and SELENOW have been described as target genes of the metal regulatory transcription factor 1 (MTF-1), but only SELENOH expression is decreased by MTF-1 e.g., in zinc-treated cells [56]. As Cu also enhances transactivation of MTF-1 [57], this could potentially be involved in the regulation of the indicated selenoprotein mRNA levels.

Second, Cu downregulated SEPSECS mRNA expression (Fig. 4B) and read-through efficiency exemplarily shown for the SECIS element of GPX4 (Fig. 4D) indicating that Cu repressed selenoprotein synthesis. SEPSECS expression has been previously shown to be decreased during acute phase response in lipopolysaccharide-treated mice [58]. Interestingly, the effects on SEPSECS and on read-through efficiency were observed in Se-treated cells only. Thus, even though the cells accumulated more Se when co-treated with Cu (Fig. 4C), they use this Se less efficiently to synthesize selenoproteins. This is also in line with the previous finding that selenoprotein mRNAs which are most sensitive towards limited Se availability (such as GPX1) mostly react on the Cu supply. Interestingly, it has been shown that remodeling of the RNA processing machinery is taking place in cells with elevated Cu [48]. Via this mechanism Cu could also interfere with selenoprotein synthesis.

The UGA recoding event is the rate limiting step of selenoprotein expression. Besides Se bioavailability, other exogenous stimuli are discussed to be modulators of Sec insertion efficiency [59]. The Cu status obviously is one of them. However, the Cu effects on read-through efficiency are mirrored only marginally on the protein levels, the third level of interaction between Cu and selenoproteins. For GPX4, a decreased expression following Cu incubation was observed under low Se conditions only, while especially GPX2 was upregulated by Cu under adequate Se conditions (Fig. 2B and C). As shown before, protein levels of selenoproteins are not very informative here because impairments of read-through efficiency could result in enhanced misincorporation of wrong amino acids instead of Sec. This effect has previously been observed after cells were treated with different antibiotics. Especially GPX1, GPX4, and TXNRD1 were highly sensitive towards replacement of Sec by cysteine or aginine [60]. Also under Se deficiency, an alternative aminoacylation of the tRNA^Ser/Sec^ with cysteine has been proposed resulting in cysteine variants of selenoproteins with lower enzymatic activity [[61], [62], [63]]. Indeed, the fourth level of Cu-induced modulation takes place at the activity level, where both total GPX and particularly TXNRD activity are inhibited by Cu. This inhibition was observed in two cell lines (HepG2 and HT29 cells) and upon co-treatment with any of two different selenocompounds, i.e., selenite and SeMet (Fig. 3A, C; S3A-D). In both cases, activity levels cannot be directly explained by changes in protein expression. All three GPXs (GPX1, 2, and 4) are unaffected or even increased by Cu under Se adequate conditions which was also the case for TXNRD1 and TXNRD2 (Fig. 2A–C, E, F). Based on this, we were wondering whether Cu can directly interfere with enzymatic assay conditions, e.g., by binding to NADPH but this was not the case, at least for the Cu concentrations that we presumably reached in our cell lysates (Fig. 3B, D). However, we could show that extracellular thiol concentrations were decreased upon Cu treatment (Fig. S1M) indicating that Cu modulates the cellular redox balance as previously extensively discussed [64]. Interestingly, previous results on the influence of Cu on GPX activity were rather heterogeneous. Similar to the data presented, treatment with 100 μM Cu showed no effect on GPX activity in HepG2 cells when no further Se was added to cells [65]. In contrast to our results, GPX activity has been reported to be decreased in Cu deficient liver and plasma of mice and rats [[23], [24], [25]]. However, in line with our results GPX activity was significantly lower in LEC rats, an animal model for Wilson's disease, with hepatic Cu accumulation in comparison to rats with lower Cu levels [66], and in Wistar rats that received an injection with Cu [67]. A lower activity of the antioxidant enzymes GPXs and TXNRDs by higher levels of Cu could contribute to Cu-induced oxidative damage and thus amplify the severity of liver disease. Also in Wilson's disease patients hepatic GPX activity is inhibited but only at stage III. The authors concluded from this result that GPX expression is first enhanced in early stages and is decreased only when the liver is severely damaged [68]. Regarding this hypothesis, we can exclude cytotoxic Cu effects for the Cu concentrations that we used herein as mechanism for the observed inhibition of GPX activity. In another clinical study on Wilson's disease, treatment naive patients were compared with patients receiving Cu reducing therapy. In those patients, higher serum Cu levels were associated with higher and not lower whole blood GPX activity [69]. For Cu effects on TXNRD activity, little data is available from the literature. But there are many well established metal- and semimetal-containing TXNRD inhibitors [70]. Here, the *in vitro* inhibition of TXNRD activity could be recapitulated in the liver of +Se/+Cu compared to +Se/-Cu mice but not in the colon though the hepatic Cu status was only very marginally affected by our intervention (Fig. 6B; 7B). Thus, Cu effects on selenoprotein activity obviously depend on multiple factors including the Cu concentration range and the organ analyzed.

**Fig. 8:**
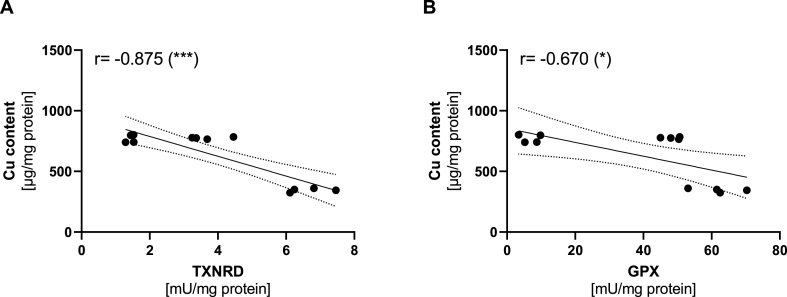
**Correlation of intracellular Cu content with enzyme activities of TXNRD and GPX.** Correlation analysis of intracellular Cu content and enzyme activity of TXNRD (A) and GPX (B) was performed using data of HepG2 cells treated with 100 μM CuSO_4_ with or without 50 nM selenite for 72 h. After 48 h of incubation, the two chelators bathocuproine disulfonic acid (BCS, 400 μM) or tetrathiomolybdate (TTM, 75 μM) were added to the cells. The TTM + Cu group was excluded from analysis because of the accumulation of non-bioactive but quantifiable intracellular Cu. *p < 0.05; ***p < 0.001 calculated based on Pearson correlation coefficient.

To mechanistically extent the *in vitro* results, we used the two Cu chelators BCS and TTM [42,71]. Two distinct modes of action may underlie the effects observed for BCS: i) Cu is chelated and retained in the media leading to a 50% reduction of intracellular Cu content (Fig. 5A), and ii) Cu is efficiently drained from inside the cell resulting in a super-depletion indicated by lower intracellular and higher extracellular Cu over time in comparison to cells without further BCS treatment during wash out (Figs. S5A and B). In contrast, TTM treatment results in higher intracellular Cu concentrations than in untreated cells [72,73]. Thus, treatment with both chelators resulted in decreased Cu bioavailability for the cells, but BCS appeared to be more efficient because the enhanced MT2a gene expression with Cu treatment was more strongly diminished with BCS than with TTM (Fig. 5B). This has previously also been shown in human neuroblastoma cells [74]. The observed effects on SEPSECS mRNA expression, GPX4 protein expression and on GPX and TXNRD activity were successfully reversed by chelator treatment and are thus Cu specific (Fig. 5D–F; S5C). In addition, there was a clear dependency of TXNRD activity and to a lesser extent for GPX activity on Cu availability. Cells with the lowest Cu availability (BCS-treated cells in combination with Cu and TTM without Cu) had the highest levels of TXNRD activity resulting in an inverse correlation (Fig. 8A and B). The correlation was stronger for TXNRD activity than for GPX activity.

In summary, Cu concentrations up to 100 μM inhibit activities of GPX and TXNRD *in vitro*. However, *in vivo* the effects were rather small under conditions of a mild modulation of the Cu status in the adequate to suboptimal concentration range in healthy, young mice. The average human serum concentration of Cu ranges from 15 to 31.5 μM [[75], [76], [77]], and thus concentrations used in cell culture experiments are adequate to supplemented. But under pathophysiological conditions up to 200 μM Cu were reported in serum [78]. In liver samples of patients with Wilson's disease or of Indian childhood cirrhosis Cu concentrations of 1.142 mg/g dry weight and 4.788 mg/g dry weight, respectively, were observed [79]. This shows that very high values of Cu can be achieved in certain diseases indicating the high relevance of our *in vitro* results. Under these conditions, not only the increase in Cu levels but a potential concomitant functional decrease of selenoproteins might by driving factors for disease progression. Also under physiological conditions, serum Cu concentrations can be increased as recently described when comparing a subcohort of the EPIC Potsdam cohort which has been reinvited after 20 years. Advanced age was associated with increased Cu concentrations and decreased Se concentrations [76]. This indicates that an age-related decline in selenoprotein expression most probably is a result of a combination of lower Se concentrations and higher Cu concentration. Also during disease, the Se to Cu ratio is frequently altered, most likely as a response to acute or chronic inflammation [80,81]. Accordingly, a higher Se intake would be needed to overcome the Cu-induced suppressive effects. These findings indicate that it is meaningful to study interactions of Se and Cu, and to understand the consequences and underlying mechanisms of this interplay in order to identify measures that may help to achieve and maintain health-supporting concentrations of these redox-relevant TEs.

## Conflicts of interest

The authors declare that they do not have any conflict of interest.

## Funding

This work was supported by the 10.13039/501100001659German Research Foundation (DFG), Germany, FOR 2558 (KI 1590/3-2).
